# Supplementary material for: Desorption of hydrocarbon chains by association with ionic and nonionic surfactants under flow as a mechanism for enhanced oil recovery
Source: Sci Rep. 2017 Aug 29;7:9586. doi: 10.1038/s41598-017-09735-8 (PMC5575256; doi:10.1038/s41598-017-09735-8)
Supplement: Supplementary file 1 — Supplementary information [file 41598_2017_9735_MOESM1_ESM.pdf]

Supplementary Information

Desorption of hydrocarbon chains by association with ionic and nonionic surfactants under flow as a mechanism for enhanced oil recovery

Ketzasmin A. Terrón-Mejía, Roberto López-Rendón
Laboratorio de Bioingeniería Molecular a Multiescala, Facultad de Ciencias,
Universidad Autónoma del Estado de México,
Av. Instituto Literario 100, 50000, Toluca, Mexico

Armando Gama Goicochea*
División de Ingeniería Química y Bioquímica, Tecnológico de Estudios Superiores de
Ecatepec, Av. Tecnológico s/n, Ecatepec, Estado de México 55210, Mexico

I. METHODOLOGY, MODELS AND COMPUTATIONAL DETAILS

A. Dissipative particle dynamics simulation (DPD)

DPD is an approach proposed to describe the primarily hydrodynamic behavior of complex fluids [S1-S3]. Based on the classical equations of motion, DPD has enjoyed enormous popularity in the modeling of systems at mesoscopic scale. DPD is a coarse-grained simulation method in which a complex molecule, such as a hydrocarbon or surfactant, is represented by soft spherical beads joined with springs. The interaction is usually described through simple and pairwise-additive potentials. Similarly to molecular dynamics simulations, particle positions and velocities in DPD are governed by the Newtonian law of motion:

$$\frac{d\mathbf{r}_i}{dt} = \mathbf{v}_i, \qquad m_i \frac{d\mathbf{v}_i}{dt} = \mathbf{F}_i, \tag{S1}$$

where $\mathbf{r}_i$, $\mathbf{v}_i$ and m_i are the position, velocity and mass of the *i*th bead, respectively, and $\mathbf{F}_i$ is the total force exerted upon it. The total force is the sum of the conservative force ($\mathbf{F}^C$),

random force ($\mathbf{F}^R$), and dissipative force ($\mathbf{F}^D$); however, given the nature of this study, an additional force was introduced, namely, an effective wall force ($\mathbf{F}^W$):

$$\mathbf{F}_{ij} = \sum_{i\neq j}^{N}\left[\mathbf{F}^C\left(\mathbf{r}_{ij}\right) + \mathbf{F}^R\left(\mathbf{r}_{ij}\right) + \mathbf{F}^D\left(\mathbf{r}_{ij}\right) + \mathbf{F}^W\right] \;. \qquad \text{(S2)}$$

The conservative force between the *i*th particle and the *j*th particle determines the thermodynamics of the DPD system and is defined by a soft repulsion:

$$\mathbf{F}_{ij}^C = \begin{cases} a_{ij}\left(1 - r_{ij}\right)\hat{\mathbf{r}}_{ij} & r_{ij} \leq r_c \\ 0 & r_{ij} > r_c \end{cases} \qquad \text{(S3)}$$

where a_{ij} is the parameter expressing the maximum repulsion between *i*th and the *j*th beads, and $\mathbf{r}_{ij} = \mathbf{r}_i - \mathbf{r}_j$, $r_{ij} = |\mathbf{r}_{ij}|$, $\hat{\mathbf{r}}_{ij} = \mathbf{r}_{ij}/r_{ij}$ is the unit vector denoting the direction from bead *i* to *j*. r_c is a cut-off radius, and it gives the extent of the interaction range between a pair of beads. The other two forces in Eq. (S2) are the random force ($\mathbf{F}^R$), which is given as follows:

$$\mathbf{F}_{ij}^R = \sigma\omega^R\left(r_{ij}\right)\xi_{ij}\hat{\mathbf{r}}_{ij} \qquad \text{(S4)}$$

and the dissipative force ($\mathbf{F}^D$):

$$\mathbf{F}_{ij}^D = -\gamma\omega^D\left(r_{ij}\right)\left[\mathbf{r}_{ij}\cdot\mathbf{v}_{ij}\right]\hat{\mathbf{r}}_{ij} \qquad \text{(S5)}$$

In Eq. (S4), σ is the amplitude of the noise. ξ_{ij} is a random number between 0 and 1 and is subject to a uniform distribution for simplicity; it is statistically independent from the pair of beads. In Eq. (S5), $\mathbf{v}_{ij} = \mathbf{v}_i - \mathbf{v}_j$ is the difference between the velocity of the *i*th bead and the *j*th bead, γ is the friction coefficient. The ω^R and ω^D are weight functions; the combination of the dissipative and random forces leads to a thermostat that conserves the total momentum of the system. The magnitude of the dissipative and stochastic forces are related through the fluctuation-dissipation theorem [S1]:

$$\omega^D\left(r_{ij}\right) = \left[\omega^R\left(r_{ij}\right)\right]^2 = max\left\{\left(1 - \frac{r_{ij}}{r_c}\right)^2, 0\right\} \qquad \text{(S6)}$$

where r_c is a cut-off distance. At interparticle distances larger than r_c, all forces are equal to zero. This simple distance dependence of the forces, which is a good approximation to the one obtained by spatially averaging a van der Waals-type interaction, allows one to use relatively large integration time steps. The strengths of the dissipative and random forces are related in a way that keeps the temperature internally fixed, $k_B T = \frac{\sigma^2}{2\gamma}$; k_B being Boltzmann's constant. The natural probability distribution function of the DPD model is that of the canonical ensemble, where *N* (the total particle number), *V*, and *T* are kept constant.

Surfactant molecules are modeled as linear chains composed of a hydrophilic head group and hydrophobic tail groups, while that hydrocarbon molecules are modeled by a neutral chain of the same type of beads. These groups are connected by a harmonic spring as follows

$$\mathbf{F}_{ij}^{S} = -k_s\left(r_{ij} - r_0\right)\hat{\mathbf{r}}_{ij} \ . \qquad \text{(S7)}$$

The spring constant is set as $k_s = 100$ and the equilibrium distance at $r_0 = 0.7$ [S4]. For simplicity, we denote water bead (W), hydrocarbon bead (HC), head group (H), and tail group (T) of the surfactant with the shorthand notations W, HC, H and T, respectively. Conservative interaction parameters for each one components are listed in Table S1. The interaction parameters have been obtained using the group contribution method (see, for example, R. Ravindra et al., *Carbohydrate Polymers* **36** (1998) 121-127) based on the solubility of each HC and following the standard technique for parametrizing the DPD interactions (R. B. Groot and P. B. Warren, *J. Chem. Phys*. **107** (1997) 4423-4435).

Within the context of DPD there are now some works that have explored the properties of fluids confined by flat surfaces. One us (AGG) proposed an effective wall potential that acts on particles close to the ends of the simulation box through a linearly decaying force [S5], in the spirit of the other DPD forces, namely

$$\mathbf{F}_i(z) = a_{w,i}\left(1 - \frac{z}{z_c}\right)\hat{\mathrm{z}}\ . \tag{S8}$$

Equation (S8) represents the force that acts on the ith-particle in the $\hat{\mathrm{z}}$ direction (perpendicular to the plane where the surfaces are placed), $a_{w,i}$ is the interaction strength of such force, and Z_c is a cutoff distance, beyond which the force becomes identically zero. A more sophisticated expression for the wall force is available (Solvation force induced by short range, exact dissipative particle dynamics effective surfaces on a simple fluid and on polymer brushes", A. Gama Goicochea and F. Alarcón, *J. Chem. Phys*. **134**, 014703 (2011). DOI: 10.1063/1.3517869), but since it raises the computational time required to reach equilibrium without adding additional insight, it was not used here.

Since in this work ionic surfactants are studied, the electrostatic interactions within the framework of DPD must be considered. For this purpose a Slater-type charge density distribution with the form of

$$f(r) = \frac{q}{\pi\lambda^3} e^{-2r/\lambda}\ , \tag{S9}$$

is adopted, in which λ is the decay length of charge q [S6]. The integration of Eq. (S9) over the entire space yields the total charge q. Charged beads interact with other charged beads through the electrostatic interaction, properly adapted for distributions of charge, such as that shown in Eq. (S9). However, they also interact with each other and with neutral beads according to their DPD interactions. Ionic surfactants are also constructed using the bead – spring model [S7], with the same parameters as those used for the non – ionic surfactants.

The efficiency of the surfactants to desorb hydrocarbons was quantified by the desorption isotherm (Γ), which represents the amount of hydrocarbon molecules adsorbed on the surface

as a function of concentration of surfactant, whether it is ionic or nonionic. Such adsorption is obtained through the following expression.

$$\Gamma = \int_0^{L_z} (\rho(z) - \rho_{bulk})\, dz \ , \tag{S10}$$

where L_z and $\rho(z)$ are the length of the cell simulation and density profile in direction z respectively, and ρ_{bulk} is bulk density, that is, the density of the unconfined system.

Finally, to model the desorption of hydrocarbons by surfactants as it is usually done in enhanced oil recovery procedures, it is necessary impart the DPD particles with an external force, so that flow in a confined channel ensues. This type of flow is known as Poiseuille flow. The method used in this work to create stationary Poiseuille flow consists of adding a constant force to all particles along the direction *x* over, as follows

$$F_x = \alpha_{flux} \ , \tag{S11}$$

where the constant α_{flux} modulates the intensity of the flow.

B. Computational details

The general conditions of simulations are described below. We use reduced units throughout this work, where all masses are taken as $m = 1.0$ and the cutoff radius is $r_C = 1.0$. The values chosen for the constants in the random and dissipative forces, σ and γ, yield $k_B T = 1$ and the time step used to integrate discreetly the equation of motion is set at $\delta t = 0.03$. All simulations are performed at constant density and temperature, i.e., using the canonical ensemble. The parameters of effective surface interaction (Eq. S8) are taken as $z_C = 1.0$, $a_{wHC} = 120.0$ (wall – hydrocarbon interaction) and $a_{wH} = a_{wT} = a_{wW} = 140.0$ (wall – surfactant Head; wall – surfactant Tail; wall – Water interaction, respectively). The solubility parameters for butane, heptane, decane and dodecane are in the range of 13.9 $(J/cm^3)^{1/2}$ to 15.5 $(J/cm^3)^{1/2}$ [S5], which yield interaction parameters $a_{ij} = 105.2, 103.8, 103.0, 102.8,$

respectively using the standard method [S2]. Therefore we opted for using an averaged value for all of those parameters, i. e. $a_{ij} = 104$, as seen in Table S1.

Three values of the external force constant necessary to create Poiseuille flow, Eq. (S11), were used, namely, $a_{flux} = 0.0000, 0.0075$ and 0.0150.

Now we describe separately each of the systems studied. In all cases, the dimensions of the simulation box are $L_x = L_y = 29.36$ and $L_z = 20.0$, in DPD units. The total number of hydrocarbon chains present in the simulation cell was equal to 180 molecules, which were adsorbed on the surfaces. This corresponds to a concentration of $C_{HC} = 0.01$. Subsequently surfactant molecules were added at increasing concentration ($C_{surfactant}$), from 0.01 to 0.10 in increments of 0.01 units.

For ionic surfactants all non-electrostatic parameters are kept equal to those of the non – ionic surfactants; the difference is that the hydrophilic groups of the ionic surfactants are assigned a charge ($q_h = 0.5e$). To keep the electroneutrality of the system, a corresponding number of counterions for every concentration of surfactant is added and the charge assigned to these counterions is $q_{ion} = -1.0e$. We employ Ewald sums to compute the electrostatic interactions, with the appropriate adaptations for confined systems using distributions of charge [S8]. The parameters of the Ewald sums are: the cutoff radius in the real space $r_{EW} = 3.5r_C$ using a $a_{EW} = 0.9695$, on the other hand in the reciprocal space we use the maximum vector $K_{max} = (5,5,5)$, and take $\beta = 0.929$ for part of charge distributions. The long surfactants (ionic and non – ionic) contain 5 beads more their short – chain counterparts. Finally, the last case of study consists of a 50:50 mixture of nonionic and ionic long chain surfactants, for every one of concentrations of surfactant $C_{Surfactant}$.

In all cases the simulations were run for 50 blocks of 10^5 time steps each, of which the first 10 blocks are used to reach equilibrium and the rest for the production phase; this is equivalent to a total simulation time of 24 μs. The total number of particles in every simulation is 50000 DPD particles with a total density of the system $\rho = 3.0$. All calculations reported in this work have been performed using the SIMES code, which is designed to study complex systems at the mesoscopic scale using graphics processors technology (GPUs) [S9].

Table S1. Conservative non – bonded, non – electrostatic DPD interaction parameters a_{ij} in units of $k_B T$. Here W, HC, H, T represent water, hydrocarbon, the head and tail of the surfactant, respectively.

	W	HC	H	T
W	78	104	39	104
HC	104	78	104	54
H	39	104	78	98
T	104	54	98	78

References

[S1] Español, P. & Warren, P. Statistical mechanics of dissipative particle dynamics. *Europhys. Lett.* **30**, 191–196 (1995).

[S2] Groot, R. D. & Warren, P. B. Dissipative particle dynamics: Bridging the gap between atomistic and mesoscopic simulation. *J. Chem. Phys.* **107**, 4423–4435 (1997).

[S3] Hoogerbrugge, P. J. & Koelman, J. M. V. A. Simulating microscopic hydrodynamic phenomena with dissipative particle dynamics. *Europhys. Lett.* **19**, 155–160 (1992).

[S4] Gama Goicochea, A., Romero-Bastida, M., & López-Rendón R. Dependence of thermodynamic properties of model systems on some dissipative particle dynamics parameters. *Mol. Phys.* 105, 2375–2381 (2007).

[S5] Gama Goicochea A. Adsorption and disjoining pressure isotherms of confined polymers using dissipative particle dynamics. *Langmuir* **23**, 11656–11663 (2007).

[S6] Ibergay, C., Malfreyt, P., Tildesley, D. J. Electrostatic interactions in dissipative particle dynamics: toward a mesoscale modeling of the polyelectrolyte brushes. *J. Chem. Theory Comput*. **5**, 3245 – 3259 (2009).

[S7] Grest, G. Normal and shear forces between polymer brushes. *Adv. Poly. Sci*. **138**, 149 – 183 (1999).

[S8] Alarcón, F., Pérez, E., Gama Goicochea, A. Dissipative particle dynamics simulations of weak polyelectrolyte adsorption on charged and neutral surfaces as a function of the degree of ionization. *Soft Matter* **9**, 3777 – 3788 (2013).

[S9] Terrón-Mejía, K.; López-Rendón, R.; Gama Goicochea, A. SIMES: SImulation at MESoscopic Scale—Accelerating Dissipative Particle Dynamics Simulations on Graphical Processing Units. In *Proceedings of the 3rd Int. Electron. Conf. Entropy Appl.*, 1–10 November 2016; Sciforum Electronic Conference Series, Vol. 3, 2016, A001; doi:10.3390/ecea-3-A001
